# Lats-IN-1 protects cardiac function and promotes regeneration after myocardial infarction by targeting the hippo pathway

**DOI:** 10.3389/fphar.2024.1463465

**Published:** 2024-10-03

**Authors:** Hua Shen, Qing Wang, Bohan Liu, Yihui Wang, Dandan Zhou, Lin Zhang, Jinqiang Zhuang

**Affiliations:** ^1^ Department of Cardiovascular Surgery, The First Medical Center of Chinese PLA General Hospital, Beijing, China; ^2^ Department of Adult Cardiac Surgery, The Sixth Medical Center of PLA General Hospital, Beijing, China; ^3^ Department of Thoracic Surgery, Shanghai Chest Hospital, Shanghai Jiaotong University School of Medicine, Shanghai, China; ^4^ Shanghai General Hospital, Shanghai Jiaotong University School of Medicine, Shanghai, China; ^5^ Department of Critical Care Medicine, Shanghai East Hospital, Tongji University School of Medicine, Shanghai, China; ^6^ Emergency Intensive Care Unit(EICU), The Affiliated Hospital of Yangzhou University, Yangzhou University, Guangling District, Yangzhou City, Jiangsu Province, China

**Keywords:** myocardial infarction, cardiac regeneration, Lats-IN-1, hippo pathway, cardiomyocyte proliferation, heart failure, apoptosis

## Abstract

**Introduction:**

Myocardial infarction (MI), a leading cause of heart failure, is characterized by the loss of cardiomyocytes, which severely limits the heart’s regenerative capacity. The Hippo pathway, which regulates cell proliferation and apoptosis, presents a therapeutic target for cardiac regeneration. This study explores the efficacy of Lats-IN-1, a LATS1/2 kinase inhibitor targeting the Hippo pathway, as a novel treatment for MI.

**Methods:**

Using male C57BL/6 mice subjected to surgically induced MI, we administered Lats-IN-1 and evaluated the effects on cardiac function, infarct size, cardiomyocyte proliferation, and apoptosis through various assays and echocardiographic assessments.

**Results:**

Our results demonstrate that Lats-IN-1 significantly improves cardiac function, as evidenced by enhanced ejection fraction and reduced ventricular dimensions. Additionally, Lats-IN-1 decreased infarct size and apoptosis rates while promoting cardiomyocyte proliferation. These findings suggest that Lats-IN-1 promotes cardiac repair and regeneration.

**Discussion:**

By modulating the Hippo pathway and reducing apoptosis markers, Lats-IN-1 represents a promising therapeutic strategy for improving outcomes in heart diseases characterized by cardiomyocyte loss. This study highlights the critical role of the Hippo pathway in facilitating cardiac regeneration.

## 1 Introduction

Cardiovascular disease remains a leading cause of morbidity and mortality worldwide, with myocardial infarction (MI) representing a major contributor to the global burden of disease (Gong et al., 2021; Zhang et al., 2023). Post-MI cardiac dysfunction is a consequence of the loss of cardiomyocytes, leading to adverse remodeling and heart failure. The mammalian heart has limited regenerative capacity; hence, there is a significant interest in identifying therapeutic strategies that can promote cardiac repair and regeneration following MI (Alonaizan and Carr, 2022).

The Hippo pathway is an evolutionarily conserved kinase cascade that plays a critical role in organ size control, tissue regeneration, and repair by regulating cell proliferation, apoptosis, and stem cell self-renewal. In the heart, the Hippo pathway restricts cardiomyocyte proliferation and regeneration, where its inhibition has been shown to enhance repair mechanisms post-MI (Aharonov et al., 2020; Ambrosini et al., 2023). Specifically, the inhibition of the core Hippo pathway kinases, such as LATS1/2, by pharmacological agents like Lats-IN-1, can activate downstream effectors YAP/TAZ, which translocate to the nucleus to drive the expression of genes that promote cell proliferation and survival (Boopathy and Hong, 2019; Cai et al., 2023).

Recent studies have provided insights into the potential therapeutic effects of targeting the Hippo pathway in the context of cardiac injury. For instance, ERBB2 has been shown to drive YAP activation and epithelial-mesenchymal transition-like processes during cardiac regeneration, signifying a link between Hippo pathway modulation and improved cardiac outcomes (Aharonov et al., 2020). Similarly, gene therapy approaches that downregulate Hippo signaling components have demonstrated promising results in inducing cardiomyocyte renewal and improving cardiac function in animal models of MI (Liu et al., 2021).

The therapeutic potential of Hippo pathway inhibition is not limited to cardiac regeneration but extends to various pathological conditions, including cancer, fibrosis, wound healing, and regenerative medicine, underscoring the versatility and significance of this signaling cascade in tissue homeostasis and disease (Dey et al., 2020). By inhibiting LATS1/2, Lats-IN-1 represents a novel approach to mitigate the post-MI inflammatory response and promote cardiac repair, as evidenced by the suppression of pro-apoptotic markers and the stimulation of regenerative pathways (Cai et al., 2023).

Given the urgent need for effective therapies to address post-MI cardiac dysfunction, this paper aims to explore the effects of Lats-IN-1 on the Hippo pathway in the context of heart regeneration. Through a series of experimental studies, we provide evidence for the beneficial role of Lats-IN-1 in enhancing cardiomyocyte proliferation, reducing scar tissue formation, and ultimately improving cardiac function after MI.

## 2 Materials and methods

### 2.1 Animals

In the context of establishing MI in animal models, male C57BL/6 mice aged 6–7 weeks were procured from the Experimental Animal Center of Shanghai General Hospital. Ethical approval for all animal-related procedures was granted by the ethics committee of Shanghai General Hospital, adhering strictly to the according to the ARRIVE guideline (https://arriveguidelines.org) and outline in the “Guide for the Care and Use of Laboratory Animals.” The mice were housed in a controlled environment at a temperature of 23°C ± 2°C with a humidity level of 50% ± 5%. The mice were randomly distributed into four distinct groups, each comprising more than five individuals: the Sham + Vehicle group, the Sham + Lats-IN-1 group, the MI + Vehicle group, and the MI + Lats-IN-1 group. To induce MI, male mice underwent tracheal intubation and were subjected to ventilation using 3% isoflurane for induction, followed by maintenance anesthesia at 2% isoflurane. Subsequently, the left anterior descending coronary artery was ligated using 7–0 prolene suture. After the surgical procedure was completed, the chest was closed, and the mice were carefully warmed until they had sufficiently recovered. The mice in the Sham + Lats-IN-1 group and the MI + Lats-IN-1 group were administered intraperitoneally with Lats-IN-1 (1 mg/kg/d) dissolved in corn oil on the first day after myocardial infarction surgery, while the mice in the Sham + Vehicle group and the MI + Vehicle group were given corn oil. Following a period of 2 weeks post-MI, the mice were anesthetized and subsequently sacrificed to facilitate the collection of heart tissues for subsequent examinations and analyses.

### 2.2 Western blot and antibodies

To analyze protein lysates obtained from heart tissue and cardiomyocytes, we utilized equal amounts of protein (30 μg) in our experiments. The protein samples were separated using SDS-PAGE and subsequently transferred onto polyvinylidene fluoride (PVDF) membranes from Merck Millipore. Following this, the membranes underwent a blocking step in which they were treated with 5% BSA in Tris-buffered saline containing 0.1% Tween 20 (TBST) for 1 h at room temperature. Next, we conducted immunoblotting by incubating the membranes with primary antibodies specific to the corresponding antigens. This incubation occurred overnight at 4°C. Subsequently, we exposed the membranes to the appropriate secondary antibodies, namely HRP-goat-anti-mouse (1:1,000, A0216, Beyotime) or HRP-goat-anti-rabbit (1:1,000, A0208, Beyotime), to facilitate binding of the primary antibodies. This secondary antibody incubation step lasted for 1 h at room temperature. To visualize the proteins on the PVDF membranes, we employed an exposure machine, specifically the GE Amersham Imager 680. We used Pierce™ ECL Plus Western blotting Substrate for protein band visualization. The quantification of protein bands was carried out using ImageJ software. For primary antibody detection, the following antibodies were employed: YAP (1:1,000, D8H1X, Cell Signaling Technology), p-YAP (1:1,000, 4,911, Cell Signaling Technology), phospho-Lats1S909 (1:500,9157), Lat1/2 (1:1,000, ab70565, Abcam), Bax (1:500, A7626, Abclonal), Caspase3 (1:1,000, 9,668, Cell Signaling Technology), Caspase9 (1:1,000, 9,502,Cell Signaling Technology). As an internal reference for normalizing protein expression levels, β-tubulin (1:2,000, ab6046, Abcam) was utilized in our experiments.

### 2.3 Histological analysis

Heart tissue underwent fixation in a 4% paraformaldehyde solution for a period of 24 h and was subsequently dehydrated using a graded ethanol series ranging from 70% to 100%. Following this, the heart tissue was embedded in paraffin. The paraffin-embedded heart blocks were transversely sectioned into 5 μm slices. After deparaffinization, the heart sections were subjected to Masson’s trichrome staining, following the instructions provided in the accompanying manual. To assess myocyte cross-sectional area, the heart sections were stained using Alexa Fluor 488-conjugated wheat germ agglutinin (WGA-Alexa488) from Thermo Fisher Scientific (Catalog No. W11261). This staining served to delineate cell boundaries and was allowed to incubate for 1 h. Subsequently, images of the stained sections were captured using a confocal laser scanning microscope, specifically the Leica TCS SP8 located in Wetzlar, Germany. The myocyte cross-sectional area and the volume of LV collagen were quantitatively measured utilizing ImagePro Plus software, version 7.0, developed by Media Cybernetics, headquartered in Rockville, MD.

### 2.4 Echocardiography

Two weeks following MI or sham surgery, echocardiographic examinations were performed on the mice. Anesthesia was induced using isoflurane (1%–1.5% for maintenance) delivered through a facemask while maintaining normal breathing. Cardiac function parameters were evaluated using long-axis M-mode echocardiography with a small animal ultrasound system (Vevo 2100, Canada) featuring a linear 30-MHz transducer. The two key indicators of cardiac function, left ventricular ejection fraction (EF) and fractional shortening (FS), were calculated by averaging measurements obtained from at least three consecutive cardiac cycles. All assessments were conducted by a single experienced technician in a blinded manner, ensuring objectivity and minimizing potential bias in the results.

### 2.5 TUNEL assay

Apoptosis was identified using the TUNEL (Terminal deoxy-nucleotidyl Transferase Biotin-dUTP Nick End Labeling) Fluorescein kit from Roche Diagnostics, based in Indianapolis, IN, and further validated by measuring cytoplasmic histone-associated DNA fragments with the Cell Death Detection ELISAPLUS kit, also from Roche Diagnostics. The method involved *in situ* labeling of fragmented DNA through tdt UTP nick end-labeling, adhering to the manufacturer’s guidelines as outlined in prior studies. The TUNEL assay, utilizing the *In Situ* Cell Death Detection Kit (Fluorescein) from Roche, enabled the detection and quantification of labeled nucleotides within nucleotide polymers through fluorescence microscopy. The average cell count was derived from five distinct high-power fields for each coverslip. The experiment was replicated three times, with each experimental condition conducted in triplicate, meaning coverslips from three different dishes were analyzed for each treatment scenario, totaling 15 fields examined.

### 2.6 Immunofluorescence

Immunostaining of 5 μm heart tissue sections and cultured cardiomyocytes involved initial steps of deparaffinization, rehydration (for heart sections), and paraformaldehyde fixation (for cardiomyocytes). Following three PBS washes, both sample types were permeabilized with 0.5% Triton X-100, followed by an additional three PBS washes. To block non-specific binding, a 5% bovine serum albumin (BSA) solution was applied for 1 h at room temperature. Subsequently, primary antibodies Aurora B (1:100, ab315206, Abcam) and Alpha Actinin (1:100, ab257317, Abcam) were incubated overnight at 4°C. After washing, secondary antibodies were applied, and DAPI was used for nuclear staining. Confocal microscopy (Leica SP8) was utilized for image acquisition. Secondary antibodies included 488-conjugated Donkey Anti-Rabbit IgG (1:1,000, AS035, Abconal) and 594-conjugated Goat Anti-Mouse IgG (1:1,000, AS054, Abconal).

### 2.7 Statistical analysis

Data analysis was performed using GraphPad Prism 8.0, and the results were expressed as mean ± standard error of standard deviation (SD). Statistical comparisons between different samples were conducted using either the unpaired Student’s two-tailed t-test or analysis of variance (ANOVA). To assess the relationship between heart fibrosis area and ejection fraction (EF) values, a correlation analysis was performed. A second-order polynomial regression model was applied to evaluate potential non-linear relationships between the fibrosis area at both the apical and papillary levels and EF values. The correlation strength and statistical significance were determined using the coefficient of determination (R^2^) and *p*-values. All statistical analyses were conducted using R software, and a *p*-value of less than 0.05 A *p*-value of ≤0.05 was considered indicative of a significant difference. It’s important to note that all experiments were repeated a minimum of three times to ensure robustness and reliability of the findings.

## 3 Results

### 3.1 Lats-IN-1 alleviates cardiac dysfunction in a MI mouse model

**FIGURE 1:**
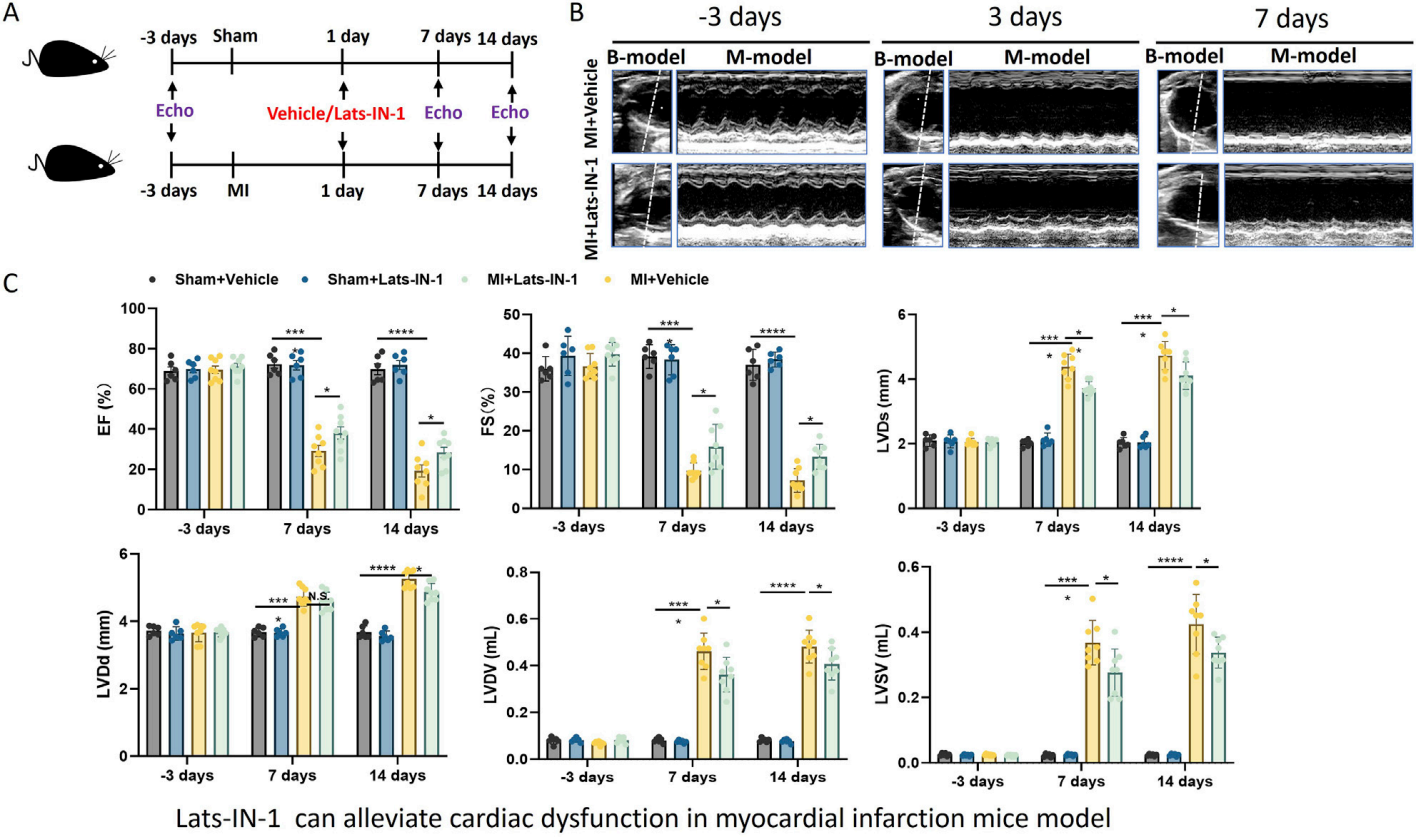
Effect of Lats-IN-1 on Cardiac Dysfunction in a MI Mouse Model **(A)**. Schematic representation of the experimental design. Mice underwent either sham operation or induction of MI, followed by treatment with either vehicle or Lats-IN-1. Cardiac function was evaluated by echocardiography (Echo) at baseline (−3 days), 1 day, 7 days, and 14 days post-MI. **(B)**. Representative echocardiographic images at −3 days (baseline), 3 days, and 7 days post-MI in mice treated with either vehicle or Lats-IN-1 post-MI, depicting the heart’s left ventricular function. **(C)**. Quantitative echocardiographic analysis of cardiac function, displayed as bar graphs. The parameters include ejection fraction (EF%), fractional shortening (FS%), left ventricular internal diameter in systole (LVIDs), left ventricular internal diameter in diastole (LVIDd), left ventricular volume in systole (LVVs), and left ventricular volume in diastole (LVVd). Data are presented for sham-operated mice treated with vehicle (Sham + Vehicle, n = 6), sham-operated mice treated with Lats-IN-1 (Sham + Lats-IN-1, n = 6), MI mice treated with vehicle (MI + Vehicle, n = 8), and MI mice treated with Lats-IN-1 (MI + Lats-IN-1,n = 8) across the time points. Data represent means ± SD. one -way ANOVA. *p < 0.05, **p < 0.01, ***p < 0.001, ****p < 0.0001; N.S. not significant.

In Figure 1, mice subjected to MI and treated with Lats-IN-1 demonstrated changes in cardiac function compared to the vehicle-treated counterparts (Figure 1A). Echocardiographic assessment showed that, at 7 and 14 days post-MI, the Lats-IN-1 treated mice had higher ejection fraction (EF%) and fractional shortening (FS%) than those treated with the vehicle. Additionally, left ventricular internal dimensions in systole (LVIDs) and diastole (LVIDd) were lower in the Lats-IN-1 group, suggesting less ventricular dilation. The left ventricular volumes in systole (LVVs) and diastole (LVVd) followed a similar pattern, with Lats-IN-1 treatment associated with smaller volumes. These changes indicate an improvement in systolic function and a potential reduction in pathological remodeling of the heart after MI in the Lats-IN-1 treated group. Measurements at the earlier time point (1 day post-MI) did not show significant differences between the treated and vehicle groups. Sham-operated mice showed no significant changes in cardiac function with Lats-IN-1 treatment when compared to sham mice treated with vehicle (Figure 1C).

### 3.2 Lats-IN-1 can reduce the area of MI

**FIGURE 2:**
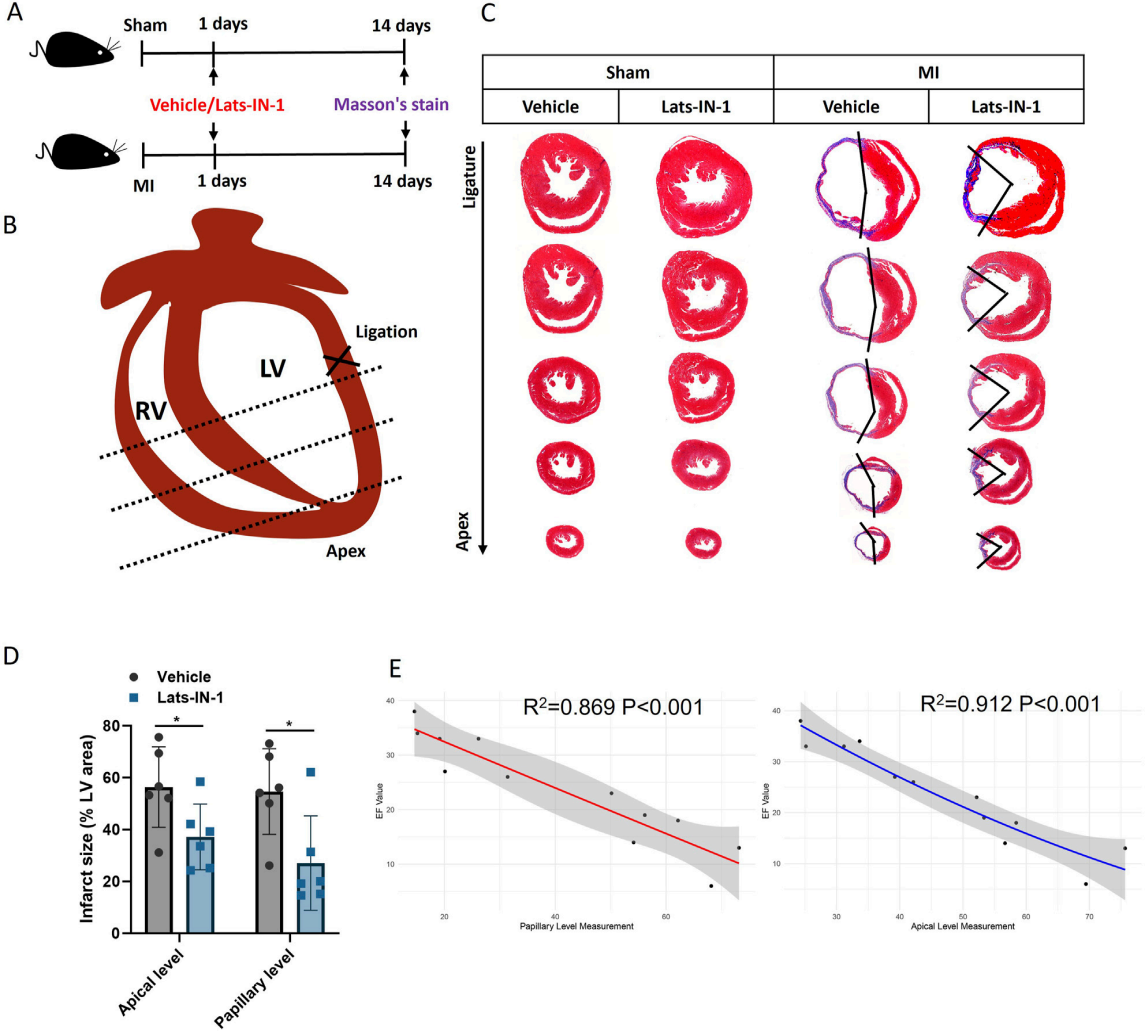
Effect of Lats-IN-1 on Infarct Size in MI **(A)** Experimental timeline indicating the procedure for sham-operated mice and MI-induced mice, both treated with either vehicle or Lats-IN-1. Masson’s trichrome staining was conducted 14 days post-MI to evaluate fibrosis. **(B)** Diagrammatic representation of a mouse heart showing the ligation site near the left ventricle (LV) used to induce MI. **(C)** Masson’s trichrome-stained heart sections from sham-operated and MI mice treated with vehicle or Lats-IN-1. The staining differentiates between viable myocardium and fibrotic tissue at the ligation and apical levels. **(D)** Quantitative analysis of the infarct size as a percentage of the left ventricular area, comparing vehicle and Lats-IN-1 treated mice at apical and papillary levels. **(E)** Correlation analysis of heart fibrosis area (papillary level and apical level) and EF value with a second-order polynomial fit. Data represent means ± SD. N = 6 for each group. Two-Way ANOVA. *p < 0.05.

Figure 2 demonstrates the effect of Lats-IN-1 on MI in mice. Masson’s trichrome staining of heart sections 14 days post-MI induction highlights fibrosis levels in sham-operated and MI mice treated with vehicle or Lats-IN-1. In the stained heart sections, the fibrotic (infarcted) areas are delineated by blue, with the remaining myocardium in red (Figure 2C). Lats-IN-1 treatment leads to a noticeable reduction in infarct size compared to the vehicle-treated group, particularly evident at both the apical and papillary levels of the left ventricle (Figure 2D). Quantitative analysis confirms a significant decrease in infarct size with Lats-IN-1 treatment, suggesting its potential to reduce fibrotic tissue formation following MI. Regression analysis in Figure 2E shows a strong inverse correlation between infarct size and ejection fraction (EF) values at both the papillary and apical levels, further supporting the efficacy of Lats-IN-1 in improving heart function post-MI.

### 3.3 Lats-IN-1 promotes cardiomyocyte proliferation in the infarct and remote zones Post-MI

**FIGURE 3:**
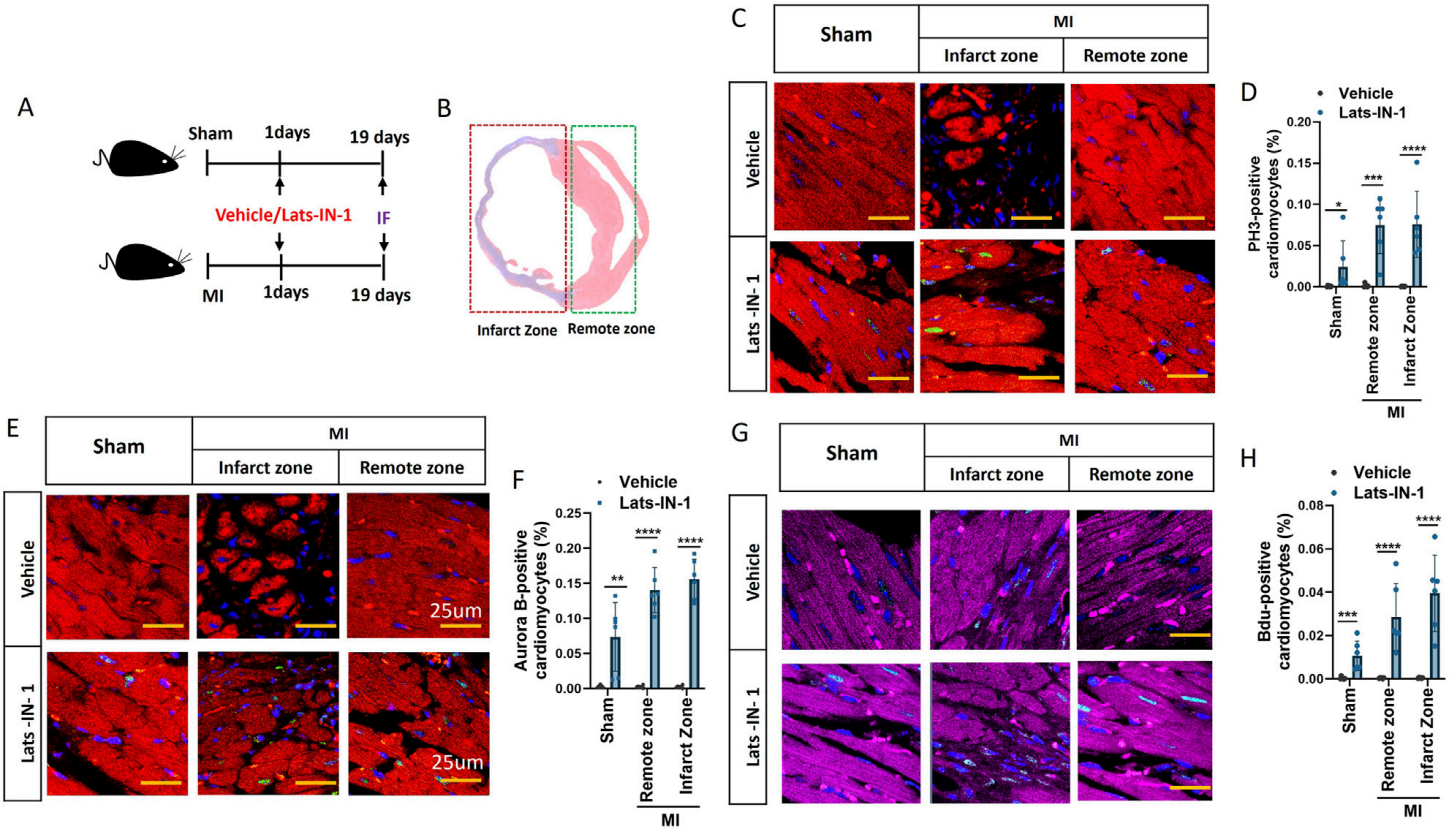
Lats-IN-1 Promotes Cardiomyocyte Proliferation Post-MI **(A)** Experimental timeline showing sham-operated and MI mice treated with either vehicle or Lats-IN-1, with immunofluorescence performed 19 days post-surgery. **(B)** Representative heart section showing the infarct zone (damaged tissue) and remote zone (unaffected tissue). **(C)** Immunofluorescence images of heart sections stained with DAPI (nuclei, blue), PH3 (proliferating cells, green), and actinin (cardiomyocytes, red), comparing vehicle and Lats-IN-1 treatment in the infarct and remote zones. **(D)** Quantification of PH3-positive cardiomyocytes as a percentage of total cardiomyocytes in sham, remote, and infarct zones. **(E)** Immunofluorescence staining of Aurora B as a marker of cardiomyocyte proliferation in the infarct and remote zones for both treatment groups. **(F)** Quantification of Aurora B-positive cardiomyocytes as a percentage of total cardiomyocytes. **(G)** Immunofluorescence staining of BrdU-positive cardiomyocytes, marking newly synthesized DNA in the infarct and remote zones. **(H)** Quantification of BrdU-positive cardiomyocytes as a percentage of total cardiomyocytes. Data are presented as means ± SD, N = 6 per group. Statistical significance: *p < 0.05, **p < 0.01, ***p < 0.001, ****p < 0.0001 (Two-way ANOVA).

We investigated the effect of Lats-IN-1 on cardiomyocyte proliferation following myocardial infarction (MI) using immunofluorescence analysis. Figure 3 illustrates that Lats-IN-1 treatment significantly increases the proliferation of cardiomyocytes in both the infarct and remote zones of the heart compared to vehicle-treated mice (Figures 3A, B). Immunofluorescence staining for PH3 (a marker of cell proliferation) and Aurora B kinase (another proliferation marker) revealed a higher number of proliferating cardiomyocytes in the Lats-IN-1 group, with notable increases observed in both the infarcted and remote regions of the heart (Figures 3C, E). Quantitative analysis confirmed a significantly greater percentage of PH3-positive (Figure 3D) and Aurora B-positive cardiomyocytes (Figure 3F) in the Lats-IN-1 treated mice. Additionally, BrdU incorporation, which labels newly synthesized DNA, further supported increased cardiomyocyte proliferation in the Lats-IN-1 group (Figure 3G), with quantification showing a significantly higher percentage of BrdU-positive cells in both the infarct and remote zones (Figure 3H).These results suggest that Lats-IN-1 enhances the regenerative capacity of cardiomyocytes in the damaged heart, promoting cell proliferation not only in the infarct zone but also in the remote, unaffected regions, thus contributing to cardiac repair after MI.

### 3.4 Lats-IN-1 can inhibit apoptosis of myocardial cells

**FIGURE 4:**
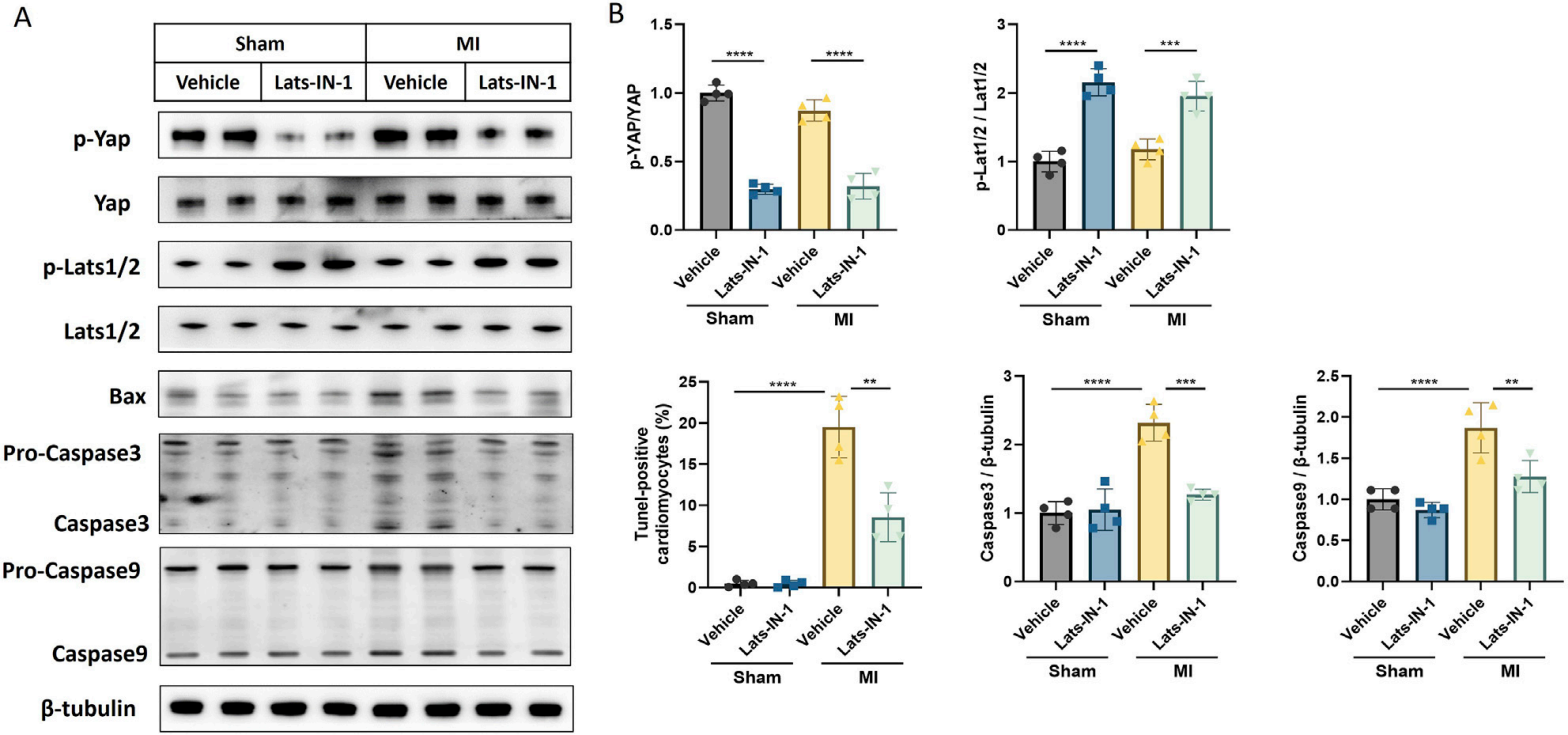
Lats-IN-1 Modulates Apoptotic and Proliferative Signaling Post-MI **(A)** Western blot analysis depicting protein expression levels of p-Yap, Yap, p-Lats1/2, Lats1/2, Bax, Pro-Caspase3, Caspase3, Pro-Caspase9, Caspase9, and β-tubulin in heart tissues from sham-operated and MI-induced mice treated with either vehicle or Lats-IN-1. **(B)** Densitometry analysis of the Western blots normalized to β-tubulin levels, comparing the expression ratios of p-YAP/YAP, p-Lats1/2/Lats1/2, Bax/β-tubulin, Caspase3/β-tubulin, and Caspase9/β-tubulin between the treatment groups. Data represent means ± SD. Two-Way ANOVA. *p < 0.05, **p < 0.01, ***p < 0.001, ****p < 0.0001.

This study investigated the impact of Lats-IN-1 treatment on various molecular markers associated with cardiomyocyte signaling and apoptosis in a MI mouse model (Figure 4). Western blot analysis shows that Lats-IN-1 treatment modulates the expression levels of key proteins in the Hippo signaling pathway. The densitometry quantification demonstrates that the phosphorylation levels of Yap (p-Yap) relative to total Yap (Yap) are reduced in the MI group treated with Lats-IN-1 compared to the vehicle-treated MI group, indicating potential activation of Yap. Similarly, phosphorylation levels of Lats1/2 (p-Lats1/2) relative to total Lats1/2 are also decreased with Lats-IN-1 treatment post-MI. Moreover, the expression of Bax, a pro-apoptotic marker, is decreased with Lats-IN-1 treatment in MI mice, suggesting a potential reduction in apoptosis. Levels of the apoptosis-executioner proteins Caspase3 and Caspase9 are also affected by Lats-IN-1 treatment, with Caspase3 showing a significant decrease in the Lats-IN-1 treated MI group compared to vehicle treatment. These results indicate that Lats-IN-1 modulates signaling pathways and apoptotic proteins in the context of cardiac injury, which may underlie the mechanisms of improved cardiac function and reduced infarct size observed with this treatment.

### 3.5 Lats-IN-1 reduces cardiomyocyte apoptosis and modulates cell size Post-MI

**FIGURE 5:**
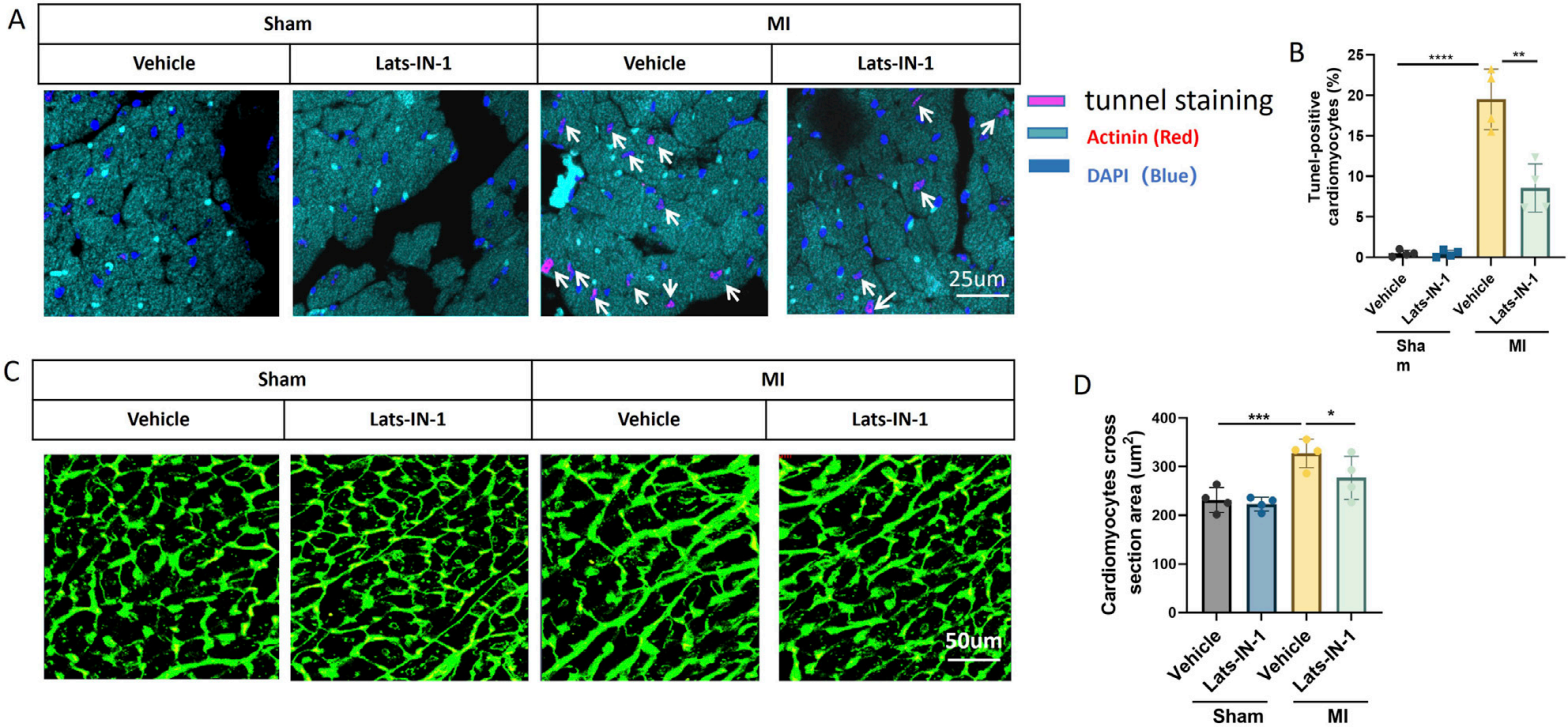
Lats-IN-1 Reduces Cardiomyocyte Apoptosis and Modifies Cardiomyocyte Size Post-MI **(A)** Representative images of heart sections from sham and MI mice treated with vehicle or Lats-IN-1, stained with TUNEL (pink) to detect apoptotic cells, actinin (red) to label cardiomyocytes, and DAPI (blue) to mark nuclei. Apoptotic cardiomyocytes are indicated by white arrows. **(B)** Quantification of TUNEL-positive cardiomyocytes as a percentage of total cardiomyocytes. **(C)** Wheat Germ Agglutinin (WGA) staining (green) of heart sections visualizing cardiomyocyte cross-sectional area. **(D)** Quantification of cardiomyocyte cross-sectional area (µm^2^) across different treatment groups. Data are presented as means ± SD, N = 4 per group. Two-way ANOVA. *p < 0.05, **p < 0.01, ***p < 0.001, ****p < 0.0001.

Figure 5 illustrates the impact of Lats-IN-1 on cardiomyocyte apoptosis and size in mice following myocardial infarction (MI). Immunofluorescence staining for TUNEL showed a significant reduction in the percentage of apoptotic cardiomyocytes in Lats-IN-1 treated mice compared to vehicle-treated MI mice, indicating that Lats-IN-1 effectively protects cardiomyocytes from apoptosis in both the infarcted and remote zones (Figure 5A). Quantitative analysis confirmed this protective effect, highlighting the potential of Lats-IN-1 in enhancing cardiomyocyte survival post-MI (Figure 5B). In addition to its anti-apoptotic effects, Lats-IN-1 treatment was associated with a decrease in cardiomyocyte hypertrophy, as evidenced by Wheat Germ Agglutinin (WGA) staining (Figure 5C). The quantification of cardiomyocyte cross-sectional area revealed that Lats-IN-1 significantly reduced the size of cardiomyocytes in the MI group compared to vehicle treatment, suggesting a beneficial effect on cardiomyocyte morphology (Figure 5D). These findings indicate that Lats-IN-1 not only promotes cardiomyocyte survival but also mitigates abnormal cell enlargement following MI, contributing to improved cardiac function and recovery.

## 4 Discussion

**FIGURE 6:**
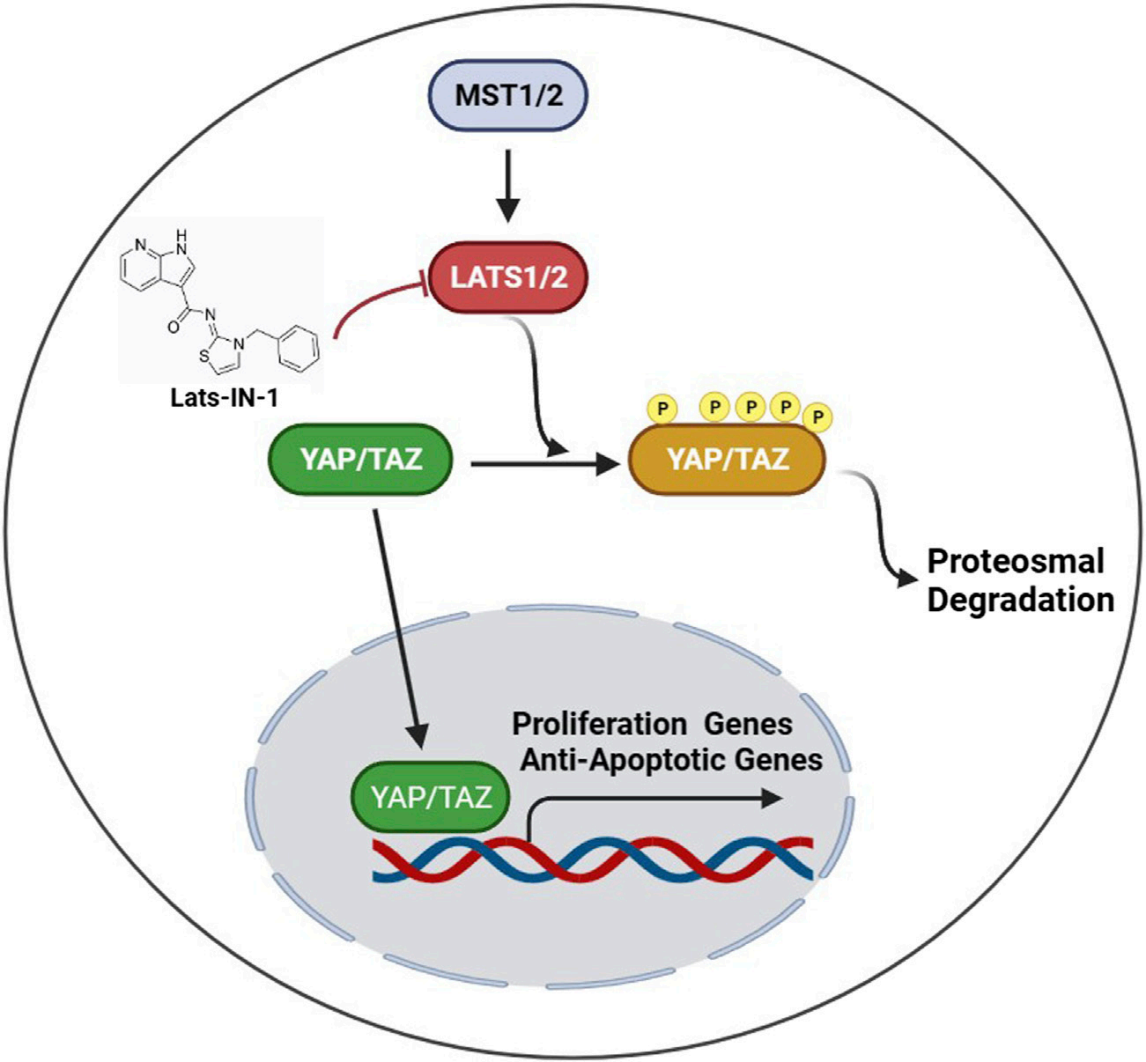
Schematic of the hippo signaling pathway and the modulatory role of Lats-IN-1.

The major findings of this study underscore the potential of Lats-IN-1 as a significant therapeutic agent in addressing MI. Our results demonstrate that treatment with Lats-IN-1 post-MI notably improves cardiac function in a mouse model. This improvement is quantitatively evident in the enhanced ejection fraction (EF%) and fractional shortening (FS%), alongside a reduction in left ventricular internal dimensions both in systole (LVIDs) and diastole (LVIDd), and in ventricular volumes (LVVs and LVVd). These changes signify not only an improvement in systolic function but also suggest a potential attenuation in pathological heart remodeling post-MI. Furthermore, the reduction in the area of MI, as indicated by Masson’s trichrome staining, implies a mitigating effect of Lats-IN-1 on cardiac fibrosis. Another pivotal finding is the promotion of cardiomyocyte proliferation in both infarcted and remote regions of the heart, as evidenced by increased Aurora B-positive cardiomyocytes. Additionally, our molecular analyses indicate that Lats-IN-1 modulates key proteins in the Hippo signaling pathway and reduces apoptosis in myocardial cells, as shown by the decreased expression of pro-apoptotic markers. Schematic illustration was shown in Figure 6.

MI remains a significant global health concern, primarily due to the adult human heart’s limited capacity for regeneration following injury (Damluji et al., 2021). MI typically leads to the loss of vital cardiac tissue and impairs the heart’s ability to pump efficiently, often progressing to heart failure (HF) (Samsky et al., 2021). The condition inflicts massive injury to the coronary microcirculation, leading to vascular disintegration and capillary rarefication in the infarct region, which exacerbates the issue by limiting blood and nutrient supply to the affected areas (Zhang et al., 2021). Consequently, the heart undergoes adverse remodeling processes, including fibrosis and changes in myocardial structure, negatively impacting its function and patient prognosis. In light of these challenges, cardiac regeneration emerges as a crucial therapeutic goal. It aims to restore the lost or damaged myocardial tissue and thereby improve cardiac function post-MI. Strategies for cardiac regeneration have primarily focused on cell therapy, employing cardiac-derived progenitor cells and engineered heart tissues. These approaches attempt to replace the cells lost after ischemic injury or to activate proliferative mechanisms within resident cardiomyocytes (Kologrivova et al., 2021). However, the complexity of cardiac tissue and the intricate interplay of various cell types involved in tissue repair underscore the need for innovative and effective strategies in cardiac regenerative medicine (Wu et al., 2021). In this context, our findings on the efficacy of Lats-IN-1 in enhancing cardiomyocyte proliferation and reducing fibrosis post-MI offer a promising avenue for future therapeutic interventions aimed at cardiac regeneration.

The Hippo-YAP (Yes-associated protein) pathway, an evolutionarily conserved regulator of organ size and growth, plays a crucial role in cardiac regeneration (Papizan and Olson, 2014). This pathway, integral to cell proliferation, apoptosis, and differentiation, presents significant therapeutic potential for various heart diseases, particularly heart failure. In cardiac development, growth, homeostasis, disease, and regeneration, the Hippo-YAP pathway is pivotal, especially in endogenous heart muscle renewal. It regulates cardiomyocyte proliferation and differentiation, essential for the heart’s response to injury, and influences the heart’s stress response and mechanical signaling. Given the human heart’s limited capacity for self-repair, particularly after MI, targeting the Hippo-YAP pathway offers a promising therapeutic approach (Mani and Diwan, 2018). Hu et al. focused on the role of fibroblast growth factor 6 (FGF6) in cardiac repair post- MI through the Hippo pathway. They demonstrated that FGF6 promotes heart function, reduces infarct size, and enhances cardiac repair by restraining the activation of the Hippo pathway and promoting nuclear accumulation of YAP, a key effector of this pathway (Hu et al., 2022). Lin et al. examined the effects of cardiac-specific YAP activation on cardiac function and survival in a murine model of MI. Their study revealed that YAP activation post-MI preserved heart function, reduced infarct size, and stimulated cardiomyocyte proliferation. They proposed therapeutic activation of YAP or its downstream targets as a potential strategy for improving outcomes after MI (Lin et al., 2014). However, our study provided a more accessible and translational way of inhibiting Hippo/YAP pathway with a small molecule Lats-IN-1.

Lats-IN-1, a LATS1/2 kinase inhibitor modulating the Hippo pathway and its downstream effects, have rarely been investigated, especially in cardiovascular research. In osteoarthritis and orthodontic research, Lats-IN-1 has been shown to significantly influence the Hippo pathway’s role in cellular response to mechanical stress. In knee osteoarthritis (KOA), mechanical loading of chondrocytes triggers the NFκB pathway, leading to increased expression of inflammatory and matrix-degrading genes. This process is regulated by the Hippo pathway, where deletion of the effector YAP or inhibition of LATS1/2 kinases using Lats-IN-1 blocks NFκB activation, thereby preventing upregulation of genes like IL1β and ADAMTS4 (Cai et al., 2023). Similarly, in human periodontal ligament cells, Lats-IN-1 was found to regulate the YAP/WNT5A/FZD4 axis, crucial for osteogenic differentiation during orthodontic tooth movement. Cyclic stretching increases WNT5A, FZD4, and nuclear YAP, with YAP positively regulating WNT5A and FZD4 expression. Inhibition of YAP alters this pathway, showing the significant role of Lats-IN-1 in modulating cellular responses to mechanical forces, thus presenting a novel perspective in treating degenerative diseases like KOA and understanding the mechanisms of bone remodeling in orthodontics (Zhang et al., 2023). Chen et al. used Lats-IN-1 to inhibit Hippo pathway and found that overexpression of DNAJB4 suppressed the adverse biological characteristics of breast cancer through modulation of the Hippo pathway and the tumor’s immune suppression context. The dose of Lats-IN-1 they used was 10 mg/kg, while we start the dose from 1 mg/kg considering different application, the side effect and the cost. The present study preliminary explored the potential effect of Lats-IN-1 in myocardium protection and heart regeneration, which showed promising results. We found that Lats-IN-1 could not only mitigate the injury induced by MI and preserve cardiac function, but also significantly promote the proliferations of myocardial cells. Limitations of this study must be noted. The study does not explore a dose-response effect of Lats-IN-1 due to limited time and resources, which is crucial for determining the optimal therapeutic dose. Without this analysis, it’s challenging to ascertain the efficacy and safety profile of Lats-IN-1 at different concentrations, which is essential for clinical application.

In summary, our study presents Lats-IN-1, a LATS1/2 kinase inhibitor, as a promising therapeutic agent for cardiac repair following MI. With further investigation, Lats-IN-1 holds promise as a novel therapeutic strategy to enhance cardiac regeneration and improve outcomes for patients with heart diseases characterized by cardiomyocyte loss and compromised cardiac function.

## Data Availability

The raw data supporting the conclusions of this article will be made available by the authors, without undue reservation.

## Abbreviations

MI, Myocardial Infarction; YAP, Yes-associated protein; LV, Left Ventricle; EF, Ejection Fraction; FS, Fractional Shortening; LVIDs, Left Ventricular Internal Dimension in Systole; LVIDd, Left Ventricular Internal Dimension in Diastole; LVVs, Left Ventricular Volume in Systole; LVVd, Left Ventricular Volume in Diastole; SEM, Standard Error of the Mean; ANOVA, Analysis of Variance; HF, Heart Failure.
