# Supplementary figures and images for: Lats-IN-1 protects cardiac function and promotes regeneration after myocardial infarction by targeting the hippo pathway
Source: Front Pharmacol. 2024 Oct 3;15:1463465. doi: 10.3389/fphar.2024.1463465 (PMC11484033; doi:10.3389/fphar.2024.1463465)

Pro-Caspase3

Caspase3

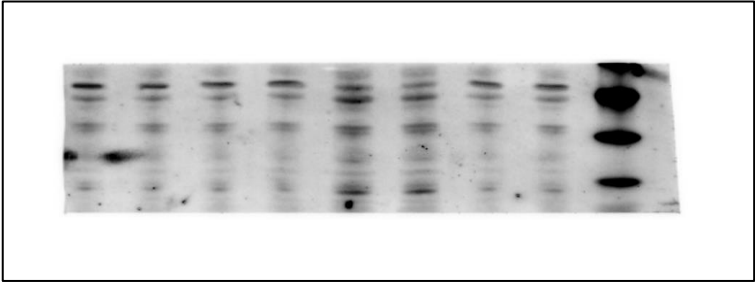

Pro-Caspase9

Caspase9

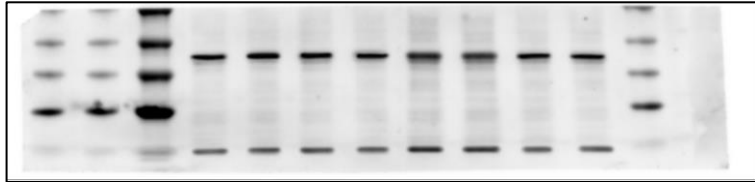

$\beta$ -tubulin

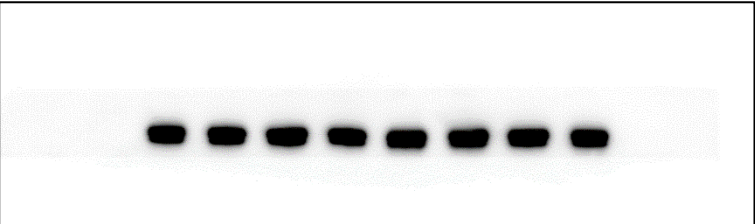

p-Yap

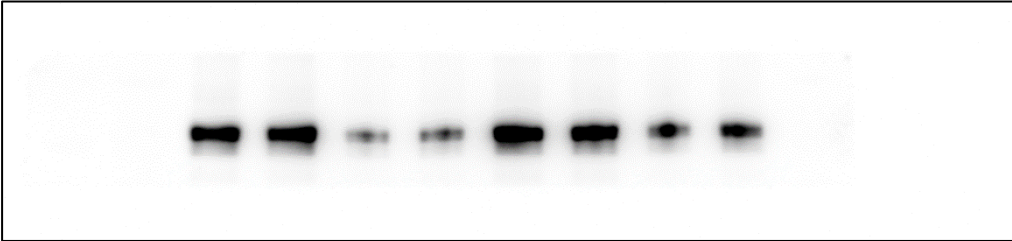

Yap

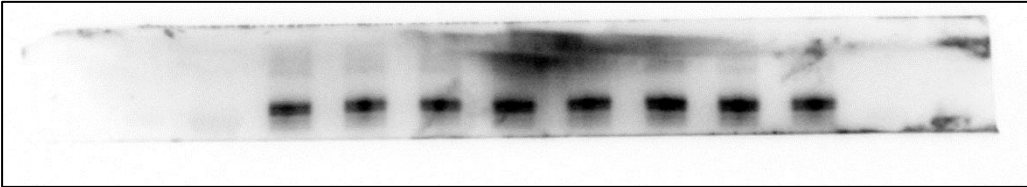

p-Lat1/2

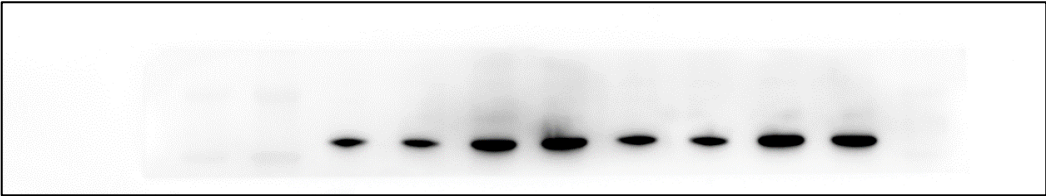

Lat1/2

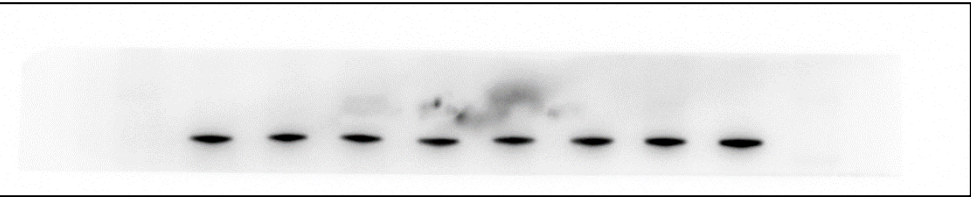

Bax

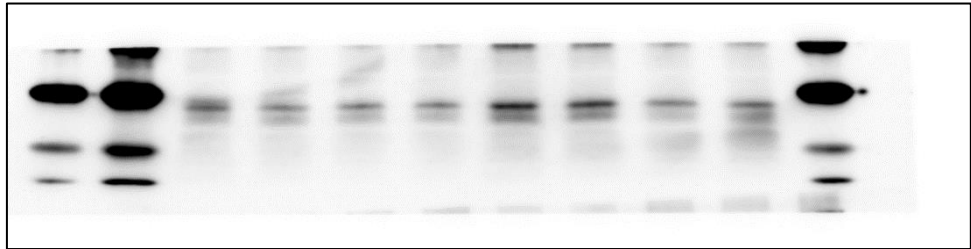

Supplement: Supplementary file 1 [file DataSheet1.PDF]
